# Use of web-based game in neonatal resuscitation - is it effective?

**DOI:** 10.1186/s12909-020-02078-5

**Published:** 2020-05-26

**Authors:** Cheo Lian Yeo, Selina Kah Ying Ho, Vina Canlas Tagamolila, Sridhar Arunachalam, Srabani Samanta Bharadwaj, Woei Bing Poon, Mary Grace Tan, Priyantha Ebenezer Edison, Wai Yan Yip, Abdul Alim Abdul Haium, Pooja Agarwal Jayagobi, Shrenik Jitendrakumar Vora, Simrita Kaur Khurana, John Carson Allen, Ereno Imelda Lustestica

**Affiliations:** 1 Department of Neonatal and Developmental Medicine, Singapore General Hospital, Singapore 169608, Singapore; 2 Yong Loo Lin School of Medicine, National University of Singapore, Singapore 119228, Singapore; 3 Duke-NUS Medical School, Singapore 169857, Singapore; 4 Lee Kong Chian School of Medicine, Singapore 308232, Singapore; 5 Department of Neonatology, KK Women’s & Children’s Hospital, Singapore 229899, Singapore

**Keywords:** Resuscitation, Newborn infants, Neonates, Memory and retention, Technology-enhanced training or learning, Serious games, Digital games, Healthcare education

## Abstract

**Background:**

Knowledge and skills decline within months post simulation-based training in neonatal resuscitation. To empower ‘Millennial’ learners to take control of their own learning, a single-player, unguided web-based Neonatal Resuscitation Game was designed. The present study investigates the effectiveness of the game on retention of resuscitation knowledge and skills.

**Methods:**

The study evaluated 162 healthcare professionals who attended simulation-based training in neonatal resuscitation. Following standard simulation-based training, participants were assigned to either a gaming group (Gamers) with access to the web-based Neonatal Resuscitation Game or a control group (Controls) with no access to the game. Although Gamers were given access, game utilization was completely voluntary and at will. Some Gamers chose to utilize the web-based game (Players) and others did not (Non-players). Knowledge and skills in neonatal resuscitation were assessed upon completion of training and 6 months post-training using a multiple-choice question test and a manikin-based skills test. Changes in scores were compared statistically between Gamers vs Controls, Players vs Controls, and Players vs Controls + Non-players using two-sample t-tests.

**Results:**

At the final assessment, declines in knowledge scores were seen in all groups. Mean change from baseline in knowledge and skill performance scores at 6 months, adjusted for baseline skill performance and MCQ test scores, did not differ significantly between Players vs Controls and Players vs Controls + Non-players.

**Conclusion:**

The web-based game in its current format may not be effective in facilitating retention of knowledge and technical skills in neonatal resuscitation.

## Background

The first minute after the birth of a newborn, also termed the golden minute is a period of anxiety for parents and healthcare providers. The newborn undergoes rapid, significant physiological changes to adapt to the extra-uterine environment [1]. Most newborns go through the transition with minimal or no assistance and initiate spontaneous respiration within 10–30 s of birth. However, approximately 10% require drying and stimulation, 3% initiate respiration after positive pressure ventilation (PPV), 2% require intubation to support respiratory function and 0.1% require chest compressions and/or adrenalin [2–4]. Failure to provide the necessary assistance at this critical time could result in birth asphyxia, with long-term complications and even death [5].

Accessibility of neonatal resuscitation is essential in all clinical settings where babies are born and cared for. Although inexpensive and cost effective, studies indicate that the need for neonatal resuscitation may not be recognised or anticipated and hence not initiated, or the methods used are inadequate or wrong [6]. Training programs in neonatal resuscitation have shown positive changes toward anticipating and preparing for neonatal resuscitation and improved resuscitation performance [7, 8]. The introduction of standardised formal neonatal resuscitation training programs has resulted in reduced early neonatal mortality in low- and middle-income countries [9]. However, knowledge and skills acquired deteriorate with time, especially when learning attained is not used or exercised for extended periods of time [10]. Mosley and Shaw [11] report deterioration of skills within 3 to 5 months post attendance at a newborn life support course.

The challenge in resuscitation retraining is to find an effective and efficient way to provide practical, hands-on experience to ensure that essential knowledge and skills are sustained once attained by an individual and/or team. Studies in computer technology-based simulation in particular games have shown acceleration of learning, increments in motivation and support for development of higher order cognitive thinking skills [12, 13]. However, the role of computer simulated games supporting self-practice in neonatal resuscitation has not been studied. Computer-based simulated games allow learning by trial and error in a risk-free environment, while maintaining a high level of realism [14].

‘Serious games’ are defined by Bergeron [15] as “interactive computer applications, with or without significant hardware components, created for the purpose of imparting knowledge or skills (such as procedural techniques, decision-making, problem-solving etc.), and which incorporate an element of scoring as well as challenging goals and engaging design” (p.122). This instructional design is known to enjoy the same advantages as simulation in that it enhances patient safety, is adaptable to specific learning objectives, standardises training at reduced operating costs and has wide accessibility [16]. Through text, sounds and pictures incorporated into the game, learning occurs as the learner reflects on an outcome relative to decisions made, performs abstract conceptualization and participates in active experimentation of complex subject matter [13, 17, 18]. Learning is reinforced with gameplay repetition as learners develop mental reasoning skills to solve unprecedented problems [19].

The opportunity to rehearse using a web-based game can potentially improve patient safety without involving the use of high-fidelity manikins and expensive simulation laboratory facilities that increase the cost of training. Evidence of possible benefits of game-based learning in healthcare education and the lack of relevant games in neonatal resuscitation to facilitate self-practice in advanced neonatal resuscitation led us to develop a single-user, unguided web-based game in neonatal resuscitation. An evaluation was performed to assess the game’s effectiveness and suitability with respect to its designated purpose and application. By measuring learning outcomes, improvements in neonatal resuscitation knowledge and skills would assure users of game viability and convince learners of the value of a web-based game as a medium for self-practice. Educators and game developers may be guided by results of outcome measures in justifying and recommending web-based games as an effective training tool. This paper reports findings of a study on the effectiveness of a web-based game as an aid for retention of knowledge and technical skills in neonatal resuscitation subsequent to ‘standard’ simulation-based training in neonatal resuscitation.

## Methods

The study is considered an educational audit and hence exempted from ethical review by the SingHealth Centralized Institutional Review Board (CIRB).

A. *Participant Selection and Intervention*

1. *Study Participants and Sampling:* Participants who attended the Singapore Neonatal Resuscitation Course (SNRC) conducted at the Singapore General Hospital (SGH) and the KK Women's & Children’s Hospital (KKH) during the period of October 2016 to July 2017 were introduced to the study at the start of the workshop, and study information leaflets detailing the study purpose and structure were disseminated. Workshop participants from October 2016 to January 2017 who consented to participate constituted the control group (Controls), and participants who attended the workshop from April 2017 to July 2017 and consented to participate constituted the experimental group (Gamers). All study groups completed the same standard, simulation-based training.

Given the lack of prior data, a ‘medium’ effect size of 0.5 was targeted as a clinically meaningful difference between the comparative groups. A sample size of 64 per group was used, based on a two-sample t-test, to detect an effect size of 0.5 with 80% power at α = 0.05 [20].

* 2) Interventions*: Gamers received access to the web-based simulation game 3 months after completion of structured training. The timeframe was decided based on a report by Mosley & Shaw [11] which showed that knowledge and skills decline as early as 3 months post-training. A user-specific password was emailed to the participants in the experimental group (Gamers) and reminders to access the game were sent monthly via email. A revised edition of the game is available at https://resuscitation.i-maginary.eu/ and details on web-based game product is attached in Additional file 1: Appendix I.

B. *Outcomes Measurements and Data Collection*

1. *Outcome Measurements and Assessment Process:* A study coordinator uninvolved in the data analysis monitored the assignment, frequency and duration of each gameplay and assisted in scheduling the follow-up assessments. Participants were not informed of the exact date of the follow-up assessment at enrolment. Email communication was made with Directors of the Clinical Departments and Nursing Division to excuse the respective study participants from their shift. To minimise disruption and inconvenience to all participants, arrangements were made in advance of the targeted test date(s). At assessments, participants complete a questionnaire detailing demographics, history of prior experience in resuscitation, and additional learning in resuscitation between training and evaluation.

A separate team of neonatal resuscitation workshop instructors not involved in the study methodology conducted the assessments. Knowledge in resuscitation was measured at two time-points using two different sets of multiple-choice questions: baseline, performed immediately post-training; and final assessment, conducted 6 months post-training. The scores from sixty MCQs were quantified and compared as a percentage of the total.

Evaluators blinded to group allocation attended standardization training on the steps, conduct and grading of the skill test prior to the start of the evaluation process. To evaluate the technical skills of neonatal resuscitation, participants were presented with a standardised scenario involving a term baby and performance in three basic key skills: 1) initial steps of resuscitation inclusive of airway manoeuvres, 2) bag-mask ventilation, and 3) chest compression were assessed using the Laerdal newborn task-trainer manikin (Laerdal Medical, Texas, USA). Baseline and final manikin-based skill tests were performed using different scenarios involving term infants. A checklist (Additional file 1: Appendixes IIa and IIb) was used to grade participants across the criteria required for the performance of key technical skills. The choice of appropriate equipment and the correct use of equipment were scored on a scale of 0 to 2. An incorrect choice or a persistently wrong application was scored as 0, and a correct choice or a consistently correction application received a score of 2. Tests were administered individually and in private by an evaluator.

*2) Data Analysis:* The baseline and final MCQ scores, and skill performance scores of Controls were collated and contrasted against that of Gamers and Players. Knowledge and skill retention was measured by differences between the mean percentage point change in MCQ scores and skill performance tests scores obtained at baseline and final assessments. Per-protocol analysis was performed to provide an estimate of efficacy of the web-based game intervention. In addition, an ‘as-assigned’ analysis comparing Players against pooled Controls and Non-players was performed.

Continuous variables were summarised as mean and standard deviation or median and range where data is skewed, and categorical variables as frequencies and percentages. In univariate analysis, mean or median values of continuous participant characteristics, MCQ and skill performance tests scores were compared between study groups using a two-sample t-test, and proportions were compared using Fisher’s exact test. Differences in MCQ and skill assessment scores were compared between Gamers and Controls after adjustment for baseline MCQ and skill performance test scores. Only participants who completed assessments at baseline and final testing were included in the final analyses. Statistical significance was set at *p* < 0.05. All analyses were performed using SAS v9.4 [21].

## Results

A. *Study Enrolment*

**Fig. 1:**
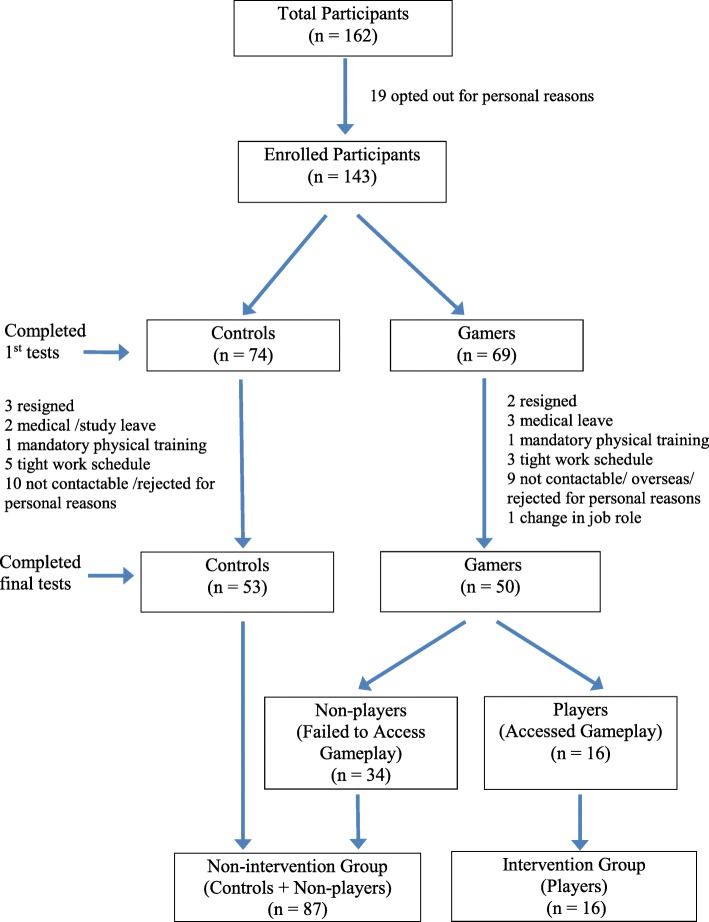
Flow diagram on enrolment of participants

In all, 162 healthcare professionals attended the course over the period October 2016 to July 2017. Upon completion of the workshop training, 143 (88.3%) of course participants (74 controls, 69 gamers) consented to participate in the study and completed the baseline assessment. Fifty-three (71.6%) controls and 50 (72.5%) gamers completed the final full tests at 6 months ±2 weeks post-training. Of the 50 experimental participants, 27 (54%) visited the game website, of whom only 16 (32%) attempted the gameplay (Fig. 1).

B. *Participant Characteristics*

1. *Control* vs *intervention based on group assignment (Controls* vs *Gamers and Controls and Non-Players* vs *Players):* There were no statistically significant differences between Controls vs Gamers and Controls + Non-Players vs Players relative to age, professional group, gender, place of work, duration of service, numbers of neonatal resuscitation attended per month or engagement in video gameplay. Of pre-existing factors that may have influenced outcomes, prior training in neonatal resuscitation - either as part of BCLS training and/or ad hoc in-house orientation at the start of their rotation, was significantly higher among Controls compared with Gamers (*p* = 0.004) (Table 1). Engagement in video games is statistically higher among Players than Controls and Non-Players (*p* = 0.001) (Table 1).

**Table 1:** Characteristics of Participants by Study Groups comparing Controls vs Gamers and Controls + Non-Players vs Players

Study groups Variables	Controls (***n*** = 74)	Gamers (***n*** = 69)	Controls + Non-players (***n*** = 87)	Players (***n*** = 16)
Professional group (%)				
- Doctors	40.5	30.4	28.8	37.5
- Nurse/Allied health staff	59.5	69.6	71.2	62.5
Age, median years (range)	31.0 (27–70)	33.0 (21–66)	32.5 (24–70)	29.5 (21–66)
Female (%)	88.6	90.0	91.2	81.3
Place of work (%)				
- NICU/SCN/Nursery	40.0	44.0	44.2	37.5
- Labour suite service	22.9	12.0	16.0	12.5
- Emergency room	5.7	–	3.0	–
- Others	31.4	44.0	36.8	50.0
Duration of service, years (%)				
- < 1	21.2	41.3	26.6	53.3
- 1–5	39.3	19.6	32.8	20.0
- 5–10	15.2	26.1	20.3	20.0
- 10-20	15.2	10.8	15.6	6.7
- > 20	9.1	2.2	4.7	–
NNR attended monthly, (n)	2.0 ± 2.4	1.6 ± 3.1	1.9 + 3.0	1.0 + 1.1
Prior NNR training -Yes (%)	17.1*	0	7.4	0
Plays video games -Yes (%)	40.0	32.7	26.9	75.0^

Table values are mean ± SD, n = numbers, percentage (%), and age in median years (range)

Prior NNR training reported consist of ad hoc in-house orientation at the start of their rotation

p = NS between study groups except where indicated

* *p*-value of 0.004 between Controls vs Gamers

^ *p*-value of 0.001 between Controls + Non-players vs Players

2) *Intervention* versus *non-intervention based on actual study participation (Players* vs *Controls, and Players* vs *Controls and Non-players)*: In evaluating the effects of the gameplay intervention on knowledge and skill retention, a per protocol analysis comparing Players vs Controls was performed. In addition, an ‘as-assigned’ analysis comparing Players vs pooled Controls and Non-players was also performed. Characteristics of Players versus Controls + Non-players were similar with median age of 29.5 years for Players compared to 32.5 years for Controls + Non-players (p = NS). The mean (SD) number of gameplay sessions performed during the intervention period preceding the final tests was 7.5 (8.1) with range of 1 to 30.

**Fig. 2:**
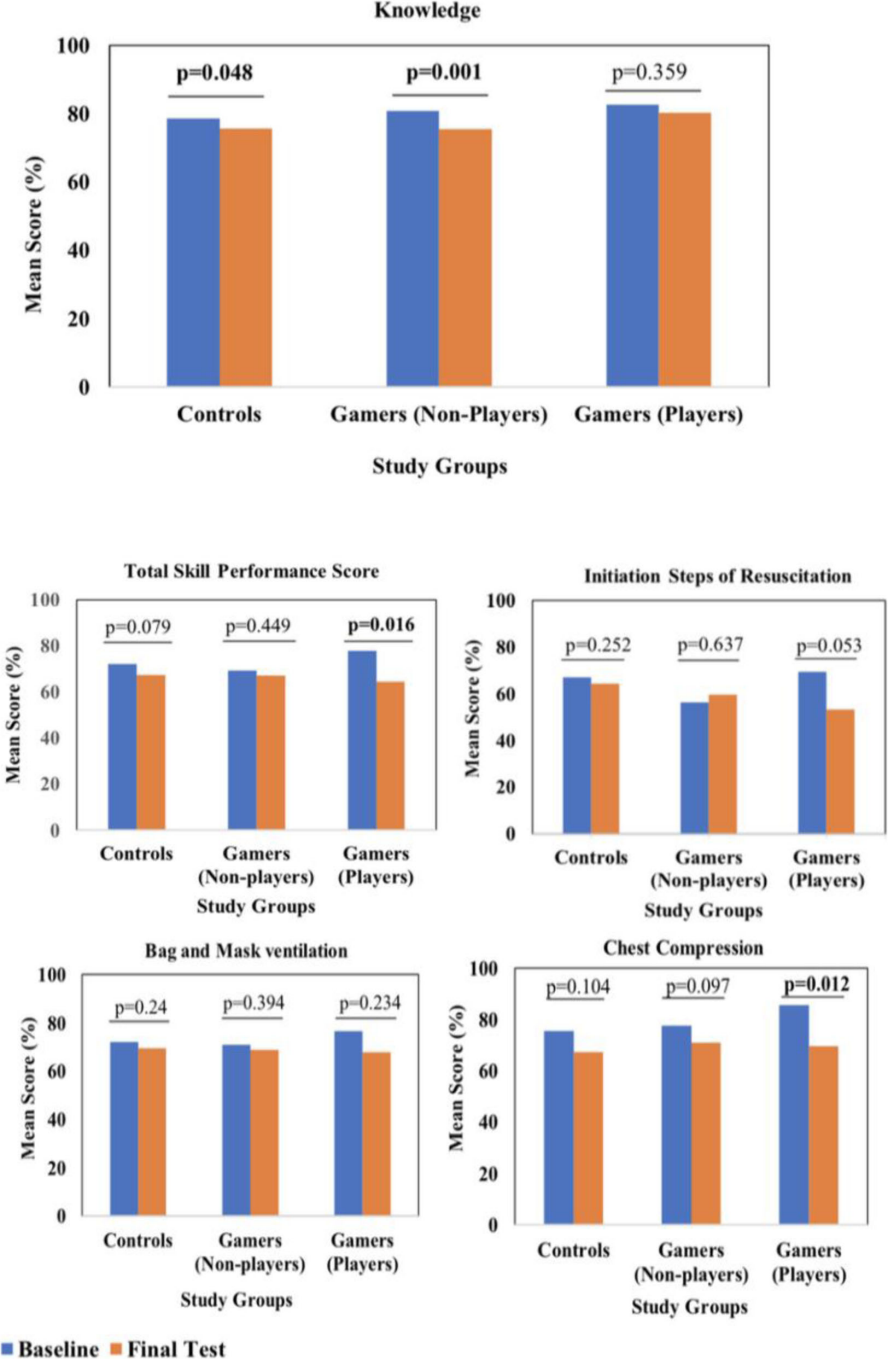
Bar Charts comparing baseline and final knowledge and skill performance test scores. Bar charts illustrates the mean knowledge, total skills and sub-skills scores at baseline and final test by study groups (Controls, Non-players and Players), *p* < 0.05 indicates significant difference in performance between baseline and final test in each study group

C. *Baseline knowledge and skill performance of study participants*: The mean (SD) baseline knowledge score for Controls was 78.6% (10.8%) compared with 81.3% (10.3%) among Gamers (*p* = 0.252). Similarly, there was no statistical difference in the baseline mean (SD) total skill performance scores between Controls and Gamers [72.1% (17.8%) versus 71.3% (16.7%)] respectively (*p* = 0.784). The Controls performed marginally better in the sub-skill of initial steps of resuscitation with a mean score of 67.1% compared to 59.4% for Gamers (*p* = 0.049), but no significant differences between groups were observed in the other sub-skills.
D. *Knowledge scores and skill performance at 6-months post-training*: A consistent decline of 2.4 to 5.8 percentage points was observed at final assessment in the knowledge scores in all study groups. The smallest decline was observed for Players (*p* = 0.357) while declines for Controls and Non-players were statistically significant with *p* = 0.049 and *p* < 0.001, respectively (Fig. 2).

Declines were observed in the skill performance scores for all study groups (Fig. 2). The most significant decline was observed for Players (*p* = 0.016) while Controls and Non-players showed smaller, non-significant decline with *p* = 0.079 and *p* = 0.449, respectively (Fig. 2). Chest compression exhibited a statistically significant decline even among Players (*p* = 0.012).

E. *Efficacy and effectiveness of web-based game on retention of knowledge and technical skills:* Per-protocol analysis measures the efficacy of the web-based game on retention of knowledge and technical skills in neonatal resuscitation. The mean knowledge test scores for Players compared to Controls were higher (80.3 ± 7.8 vs 75.7 ± 11.3, *p* = 0.072) at final assessment but there was no difference in change in mean knowledge test scores from baseline to the final assessment (− 2.4 ± 10.1 vs − 2.6 ± 9.3, *p* = 0.949) for Players compared to Controls (Table 2). After adjusting for baseline knowledge level, a smaller non-significant decline in mean knowledge test scores was noted for Players compared to Controls (Table 2). At baseline assessment, the mean total skill performance scores of Players was higher at 77.8 + 15.8 compared with 71.7 + 17.3 in the Controls (*p* = 0.192), with statistically better performance at chest compression (85.7 + 14.8 vs 73.7 + 21.7, *p* = 0.016). The decline in total skill and sub-skills performance scores at final assessment was greater in the Players compared to Controls with respective declines of 9 to 16 versus 3 to 7 percentage points (Table 2). Adjusting for baseline skill levels, the decline in total skill and sub-skills performance scores from baseline to final assessment remained greater for Players compared with Controls (Table 2). Per-protocol analysis reflects a decline in technical skills of resuscitation in the Players but bias due to exclusion of participants who did not access gameplay cannot be ruled out. The ‘as-assigned’ analysis comparing Players vs Controls + Non-players yielded similar changes in knowledge, total skills and sub-skill performance from baseline to 6 months post-training (Table 3).

**Table 2:** Comparison of knowledge and skill performance scores at 6 months between Players vs Controls

Scores (%) Tests	Mean Change from BL (unadjusted)			Mean Change from BL (adjusted)^**a**^		
	Controls (***n*** = 53)	Players (***n*** = 16)	***p***-value	Controls (***n*** = 53)	Players (***n*** = 16)	***p***-value
Knowledge	−2.6 ± 9.3	−2.4 ± 10.1	0.949	−3.3 ± 8.55	−1.2 ± 6.93	0.331
Total Skill Performance	−4.3 ± 17.6	−13.3 ± 19.5	0.115	− 4.5 ± 16.0	−10.0 ± 16.2	0.250
Initial Steps of Resuscitation	−3.3 ± 27.6	−15.9 ± 30.3	0.149	−0.3 ± 20.6	−11.6 ± 21.5	0.078
Bag & mask Ventilation	−3.1 ± 25.6	−8.8 ± 28.3	0.485	−3.6 ± 22.3	− 6.5 ± 24.2	0.673
Chest Compression	−6.5 ± 31.1	− 16.1 ± 22.4	0.181	−8.0 ± 26.8	−10.7 ± 19.3	0.659

^**a**^Adjusted for baseline (BL) MCQ and skill performance test scores

Table values are mean ± SD, and n = numbers as indicated

**Table 3:** Comparison of knowledge and skill performance scores at 6 months between Controls + Non-players vs Players

Scores (%) Tests	Mean Change from BL (unadjusted)			Mean Change from BL (adjusted)^**a**^		
	Controls + Non-players (***n*** = 87)	Players (***n*** = 16)	***p***-value	Controls + Non-players (***n*** = 87)	Players (***n*** = 16)	***p***-value
Knowledge	−3.8 ± 9.3	−2.4 ± 10.1	0.575	− 4.0 ± 8.2	− 1.2 ± 8.2	0.213
Total Skill Performance	−3.6 ± 18.1	−13.3 ± 19.5	0.055	− 4.2 ± 15.5	−10.0 ± 15.5	0.179
Initial Steps of Resuscitation	−1.0 ± 28.3	−15.9 ± 30.3	0.059	− 1.8 ± 20.7	−11.6 ± 20.7	0.086
Bag & mask Ventilation	−3.4 ± 25.3	−8.8 ± 28.3	0.443	− 3.8 ± 21.5	−6.5 ± 21.5	0.649
Chest Compression	−5.9 ± 26.4	− 16.1 ± 22.4	0.152	− 7.0 ± 22.5	−10.0 ± 22.5	0.634

Table values are mean ± SD, and n = numbers as indicated

^**a**^Adjusted for baseline (BL) MCQ and skill performance test scores

Modified intention-to-treat analysis (ITT) including study participants who completed both the baseline and final tests showed that the use of web-based game resulted in no significant differences in the mean knowledge test scores and technical skill performance scores, both total and sub-skills at final assessment between the Gamers (Players and Non-players) and Controls (Table 4). After adjusting for baseline skill performance levels of participants, the change in total skill and sub-skill performance from baseline to 6 months post-training in both Controls and Gamers exhibited no statistically significant differences.

**Table 4:** Comparison of knowledge and skill performance scores at 6 months between Controls vs Gamers (Players + Non-players)

Scores (%) Tests	Mean Change from BL (unadjusted)			Mean Change from BL (adjusted)^**a**^		
	Controls (***n*** = 53)	Gamers (***n*** = 50)	***p***-value	Controls (***n*** = 53)	Gamers (***n*** = 50)	***p***-value
Knowledge	−2.6 ± 9.3	− 4.7 ± 9.37	0.252	− 3.3 ± 8.55	− 3.99 ± 8.29	0.653
Total Skill Performance	− 4.3 ± 17.6	− 6.0 ± 19.7	0.662	− 4.5 ± 16.0	− 5.79 ± 15.6	0.675
Initial Steps of Resuscitation	−3.3 ± 27.6	−3.5 ± 30.7	0.973	− 0.3 ± 20.6	− 6.63 ± 20.7	0.127
Bag & mask Ventilation	−3.1 ± 25.6	− 5.3 ± 26.0	0.668	−3.6 ± 22.3	−4.90 ± 21.5	0.753
Chest Compression	−6.5 ± 31.1	−8.6 ± 19.5	0.684	−8.0 ± 26.8	−6.72 ± 22.5	0.739

Table values are mean ± SD, and n = numbers as indicated

^a^Adjusted for baseline (BL) MCQ and skill performance test scores

## Discussion

Our study demonstrated significant declines in both knowledge and technical skills at 6 months following a structured neonatal resuscitation course attended by participants assigned to control and web-based game intervention. In the control group, it was observed that technical skills encompassing total performance and sub-skills exhibited greater declines on average (− 3.1 to − 6.5 percentage points) than theoretical knowledge (− 2.6 percentage points) 6 months after standard simulation-based training. Dunn et al. [7] compared knowledge and skill levels in 166 nurses at initiation and after 6 months. At the 6-month follow-up, none of the participants in either groups, ‘controls’ or ‘trained’, passed the skill test, while 85% of the ‘trained’ group obtained a passing mark of 80% on MCQ tests, compared to 23% of the controls, suggesting faster deterioration in skills than in knowledge.

Our findings of diminished retention of knowledge and technical skills in all study groups over time are consistent with the literature. Although all studies differed with respect to the targeted population, the format of initial neonatal resuscitation training, and in measurements and analytical methods on outcomes studied, retention of knowledge and skills exhibited similar patterns of decline within 3 to 6 months after attendance of structured formal training. Differences in degree of decline in retention among studies can be explained by variation in study populations, differences in clinical practice among study populations post-training, and variation in criteria used at testing.

A. *Effect of web-based game on retention of knowledge*

In our study, knowledge in all groups declined over the 6-month study period. Despite higher final mean knowledge scores for Players compared to Controls (*p* = 0.072), and Players compared to Controls + Non-players (*p* = 0.046), the changes in mean knowledge scores from baseline to final evaluation in the Players compared with (i) Controls and (ii) Controls + Non-players were not statistically different, suggesting that web-based game in its current format may not be efficacious for knowledge retention in resuscitation. The knowledge deterioration following initial learning plateaued with the introduction of gameplay. However, it is not clear if longer-term practice using the neonatal resuscitation web-based game can improve retention of knowledge in neonatal resuscitation.

There are no comparable serious games that teaches advanced neonatal resuscitation. Video games and VR games developed to reinforce theoretical and basic neonatal resuscitation skills have evaluated learner’s attitudes toward the game but report on educational and clinical outcome is lacking. Of 14 nursing students enrolled to e-baby, a computer-based game designed to teach the assessment of oxygenation in preterm babies for 2 weeks, 57% reported game to be easy to use with 72% motivated to use it for learning [22, 23]. Umoren, Bucher, Purkayastha, Kshatriya, & Avanigadda [24] evaluated the use of Electronic Helping Babies Breathe (eHBB), a computer game developed to practise knowledge in neonatal resuscitation and skills on administering PPV on 24 healthcare workers in resource-limited settings reported that game was educational, easy to use and enabled ‘learning without stress’. Umoren et al. [25] evaluated the use of NRP e-SIM, a simulator game developed to reinforce cognitive skills and the steps in neonatal resuscitation before a hands-on NRP course [26, 27] in a randomised trial involving 255 NRP providers. Findings showed better accuracy in the performance of several steps of the NRP algorithm in the group randomised to pre-NRP course preparation of e-SIM + NRP textbook compared to the textbook only group. However, there was no difference in time needed to perform key NRP steps inclusive of time to start PPV and chest compressions. The effect of repeated e-SIM practice on knowledge and or skill retention was not addressed in the study.

However, studies on the associations between hours of simulation-based practice in Paediatric cardiopulmonary resuscitation training and learning outcomes indicate a dose-response relationship, with more practice producing higher outcome gains and minor variation among learners in time required to achieve a level of mastery [28–30]. Findings showed that the percentage of healthcare provider performance rated ‘excellent’ increased from 26 to 65% with use of low dose, high frequency booster training [29]. A study that compared 6 min of monthly practice on a voice advisory manikin to no practice showed that monthly practices resulted in improved skills over baseline [30]. It is also unknown whether repeated tests over a longer duration of gameplay would have a longer-term impact.

The modified ITT analysis comparing final assessment scores showed no difference in change in mean knowledge score from baseline to 6 months for either Controls or Gamers (Players and Non-players), suggesting that the web-based game, as presented to the study participants may not be effective in motivating self-practice or increasing knowledge retention in neonatal resuscitation.

B. *Effect of web-based game on Skill Performance*

In this study, the total skill performance and performance at chest compression were statistically significantly lower at final test for Players compared to Controls and compared to Controls + Non-players. Modified ITT analyses showed similar patterns of decline in performance scores in both total and sub-scales in both the Control and Gamers groups, with the larger decline from baseline to final test in the Players sub-group (p = NS). These tests were conducted using low-fidelity manikins, hence it may be more difficult for the participants to suspend disbelief compared to a real-life situation. However, both the baseline and final tests were done using similar manikins. A study by Campbell, Barozzino, Farrugia, & Sgro [31] that compared the use of low and high fidelity on knowledge and skill acquisition pre- and post-training showed a greater but statistically non-significant improvement in written test scores and shorter times to intubation in the group trained using the high-fidelity manikin. Hence, it remains unclear whether the use of a low-fidelity manikin influences scores in this study.

There is no comparative game in neonatal resuscitation, but a report on the use of MicroSim, a computer game that simulates medical emergencies and procedures designed to support retraining in Advanced Life Support showed no impact on retention of skills [32]. In the study, 20 % of participants elected not to use the intervention program during the one-year study and of those who accessed the MicroSim program, less than 50 % of the users completed the program. Study participants reported lack of social interaction and the lack of motivation to use the tool as reasons for the absence of a significant effect with the use of MicroSim.

Social interaction during training facilitates learning and sharing of resources [33], provides opportunities for learners to draw on their peers to fill gaps in their own knowledge [34], provides learners with emotional support, and makes learning more active and engaging [35, 36]. Our single-user, unguided web-based game provides no opportunity for collaborative interaction, hence missing in extrinsic motivation. Motivation has several effects on learning and learner behaviour. A motivated learner is more likely to initiate and persevere at a task, increasing effort for activities related to their goals [37] and better performance. Elements of fun, challenge, feedback, and rewards embedded in a web-based neonatal resuscitation game can entice and sustain users at gameplay repeatedly and for prolonged sessions, leading to better performance.

The significant decline in mean percentage scores in the total and sub-skill performance tests among participants who practiced using the web-based game suggest that not only is retention absent, but exposure to gameplay may have resulted in negative transfer of skills, hence interfered with skill performance at retest [38]. Negative transfer occurs when previous learning or experience inhibits or interferes with performance in a new context [39]. This may occur in a situation where differences or discontinuity in terms of spatial location and timing of movements between practice using web-based game scenario and the ‘real’ scenario occurs, and game users may modify their performance in a negative way. In addition, if game users have made a connection between skills viewed in the game and they differ from skills that are required in real life, the result will be negative transfer. These two factors can occur using games to train skills in resuscitation, hence accounting for the inappropriate transfer of skills associated with gameplay. In our study, negative transfer noted in the small cohort of 16 participants raises concern but in the absence of statistical significance, the results cannot not be generalised. Further investigation involving a larger sample size is needed.

C. *Study Limitations and Strengths*

The use of controls, standardised MCQs and manikin-based tests, blinding of evaluators and standardisation of skill evaluation among evaluators strengthen the scientific and statistical validity of our study.

Although the demographics of the study groups are similar, unrecognised differences in clinical practice and variations in exposures to sentinel events involving newborn resuscitations among study participants between the two time-points (i.e. baseline and final evaluations) may occur leading to change in resuscitation practices, hence confounding outcomes measured.

The high drop-out rate of 28% in all study groups at final evaluation led to a reduction in sample size. With only 23% of the enrolled cohort accessing the game, the power of the study declined leading to risk of a type 2 error. Next, measuring the learning outcomes at a single time-point of 6 months post-training further restricted the observation on skill performance and MCQ tests. In addition, the MCQ tests and the checklist used were not formally validated. Factors such as learner motivation to train using this approach, variation in entry levels of participant skill in the use of computer-based technology and resource use that may interfere with the outcomes measured were also not evaluated in our study.

## Conclusions

In summary, this study reaffirms results of previous studies that knowledge and technical skills associated with neonatal resuscitation decline 6 months post-training, hence practice and more frequent retraining is necessary to maintain knowledge and technical skills in neonatal resuscitation. The evaluation suggests that the game in its current format and making it available to learners 3 months after the standard simulation-based training on a voluntary basis may not be effective in supporting self-practice or in aiding retention of knowledge and technical skills in neonatal resuscitation. However, given the relatively low statistical power and internal validity concerns of the study, the statistical evidence supporting conclusions about the relationships between use of the web-based game and its effect on knowledge and skill retention is weak [40]. More evidence is needed for a complete evaluation of game effectiveness.

## Supplementary information

**Additional file 1: Appendix I.** Details of Web-based Game Product. **Appendix II** a. Skills Assessment Checklist (Baseline Assessment). b. Skills Assessment Checklist (Final Assessment).

## Data Availability

The datasets generated and analysed during the study are not publicly available as approval is not currently available from the Health Service Research Unit of SingHealth, the local health cluster where study is conducted. However, datasets are available from the corresponding author on request.

## Abbreviations

BCLS: Basic Cardiac Life Support
BL: Baseline
CIRB: Centralized Institutional Review Board
ITT: Intention-to-treat
MCQ: Multiple-choice question
NICU: Neonatal Intensive Care Unit
NNR: Neonatal resuscitation
NRP: Neonatal Resuscitation Program
NS: Not significant
PPV: Positive pressure ventilation
SCN: Special Care Nursery
SD: Standard deviation
