# Supplementary material for: Use of web-based game in neonatal resuscitation - is it effective?
Source: BMC Med Educ. 2020 May 26;20:170. doi: 10.1186/s12909-020-02078-5 (PMC7249390; doi:10.1186/s12909-020-02078-5)
Supplement: Supplementary file 1 — Additional file 1: Appendix I. Details of Web-based Game Product. Appendix II a. Skills Assessment Checklist (Baseline Assessment). b. Skills Assessment Checklist (Final Assessment). [file 12909_2020_2078_MOESM1_ESM.docx]

Appendix I

**Details of Web-based Game Product**

A synopsis of the game is available at: <https://www.youtube.com/watch?v=0aDzjJTWUsc>. Players are able to access the revised edition of the game via the website at [https://resuscitation.i-maginary.eu/](https://imsva91-ctp.trendmicro.com/wis/clicktime/v1/query?url=https%3a%2f%2fresuscitation.i%2dmaginary.eu&umid=9797FCDE-9CB7-A805-BB30-06042CADAB5D&auth=6e3fe59570831a389716849e93b5d483c90c3fe4-8c4e8f5ee2adc6ac91a49fd445ed64313c84dea9) . On logging using user-specific id and password, the main screen with the game logo appears as shown in the image 1. At the start, player may select to play a preferred scenario or access the help menu where tutorial videos on layout and format of the game are available (image 2). Upon selection of the preferred scenario, details pertaining to the case will be presented (image 3). A virtual dialogue is created between the player and the attending nurse to provide her/him with the necessary information to facilitate preparation of equipment (image 4). Dialogue is created with the support of a list of 20 Multiple-choice question statements from which the player selects the 5 relevant questions (gestational age, estimated foetal weight, clarity of amniotic fluid, numbers of babies, and other maternal risk factors). Three non-player characters are available to support in resuscitation. The player serves as the team-lead. She/he chooses the appropriate therapeutic treatments and delegates the tasks accordingly (image 5). Interventions are time-specific and accuracy in sequence of steps monitored. The goal in each scenario is to achieve heart rate of more than 100 beats per minute with appropriate oxygen saturation. Breathing may be supported via positive pressure ventilation delivered via endotracheal tubes (ET) or continuous positive airway pressure (CPAP) delivered via nasal CPAP prongs or mask depending on the breathing effort of the baby. At the end of the gameplay, feedback with scores measured in term of percent accurate as determined by the effectiveness of PPV (either ET or face-mask), technique of chest compression and attention at coordinating both tasks for 60 seconds are presented to the player (image 6).


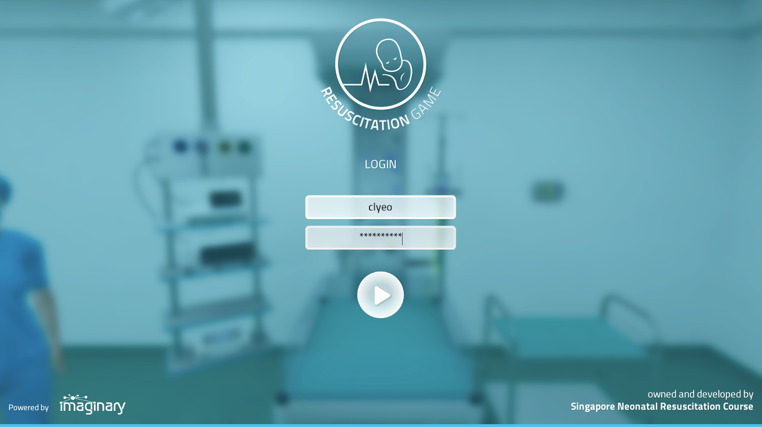
 Image 1 – Page on log-in


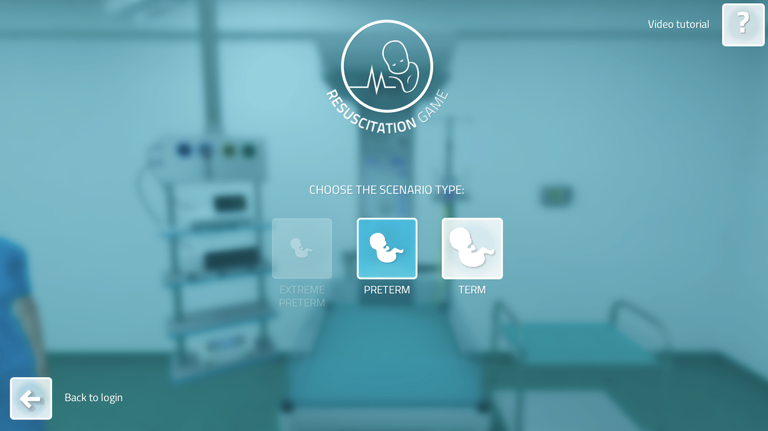
 Image 2 – Scenario selection


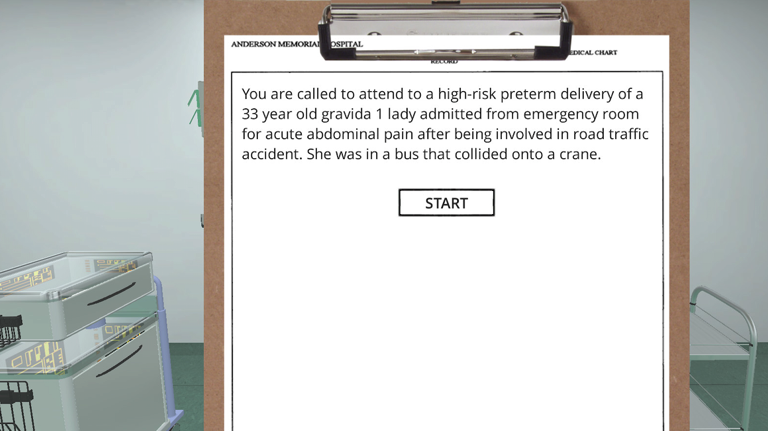
 Image 3 – Scenario case summary


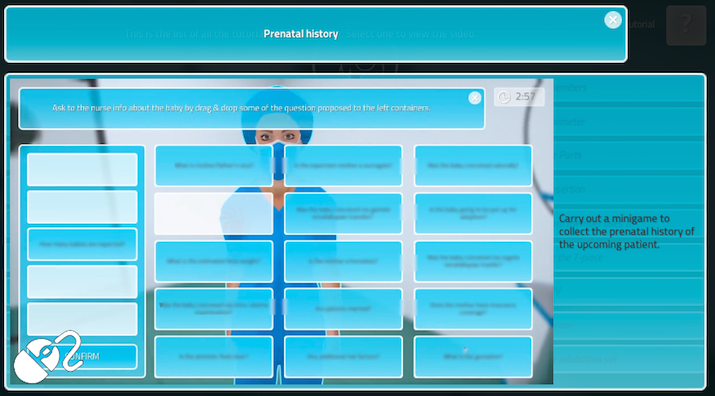
 Image 4 – Dialogue for case history


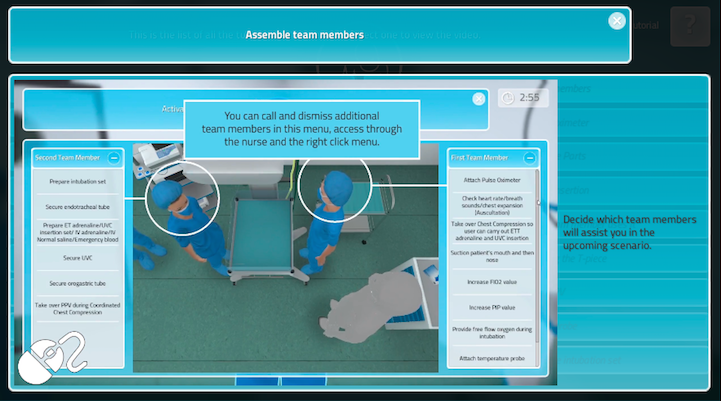
 Image 5 – Role delegation


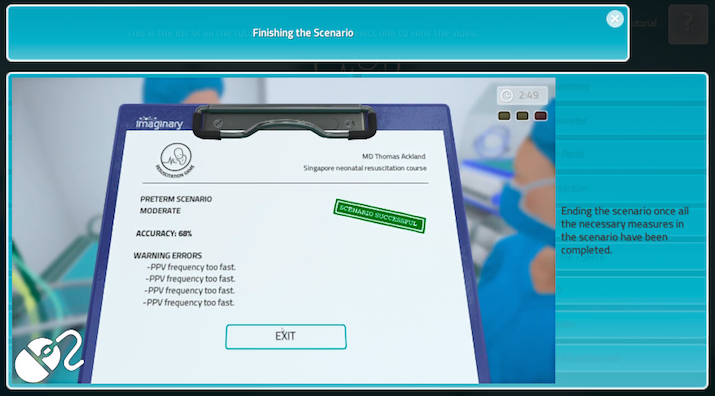
 Image 6 – Feedback

Appendix IIa

**Skills Assessment Checklist (Baseline Assessment)**

Participant ID/Name: _________________________ Date of Assessment: ____________

Evaluator ID/Name: __________________________

**Scoring: 0**=Not Done **1**=Done incorrectly, incompletely or out of order **2**=Done correctly in order

| ***“As part of our evaluation of the e-simulated learning tool, we are reviewing whether the tool improves the key basic skills of neonatal resuscitation. When you begin the resuscitation, work as if this were a real baby & a real resuscitation. The mannequin does not have a pulse or change colour, so please ask me for additional information. If you require equipment beyond what is available to you, do inform us. If you require an assistant, indicate that you need help. I will be the assistant but please tell me what to do. Do you have any questions?”*** | | | |  |
| --- | --- | --- | --- | --- |
| **Scenario** | | | |  |
| ***“You are called to attend an unplanned delivery in the antenatal ward. The mother is in active labor with ruptured membranes. It is a singleton pregnancy at 39 weeks. Mother has been admitted in the ward for one week for Pregnancy Induced Hypertension and is on medication. There are no other risk factors and the amniotic fluid is clear.”***  ***“The equipment has been checked.” (Evaluator to orientate participants on equipment available)***  ***“The baby is born.”*** | | | |  |
| **Skills** | **0** | **1** | **2** |  |
| 1. **Initial Assessment & Initial Steps** | | | |  |
| Start timer  **0**=Not started / Started late, **1**= Started immediately |  |  |  |  |
| 4 questions - Term, Liquor, Tone, Crying/Breathing?  **0**=Does not ask Tone & Crying/Breathing, **1**=Ask at least Tone & Crying/Breathing, **2**=Ask all 4 |  |  |  |  |
| ***“ Term, Liquor clear, Limp, Apnoeic”*** | | | |  |
| Dry baby, Keep warm and Remove wet towel  **0**=Not done, **1**=Incomplete drying, not removed wet towel, **2**=Perform both  (*No penalty for not mentioning “pre-warmed’*) |  |  |  |  |
| Position airway  **0**=Neck flexed / hyperextended, **2**=Correct position |  |  |  |  |
| Suction mouth & nose  **0**=Not done, **1**=Incorrect order, duration, depth, **2**=Correct order, duration, depth |  |  |  |  |
| Stimulate by flicking soles &/or rubbing back  **0**=Not done / incorrect, **1**=Stimulate correctly |  |  |  |  |
| Evaluation: Respiration, HR**0**=No/improper evaluation/Only 1, **1**=Both (*No point for HR assessment via umbilical pulsation*) |  |  |  | **Subtotal**  11 |
| ***“Apnoeic, HR = 80/min”*** | | | |  |

| 1. **Bag & Mask Ventilation** | | | |  |
| --- | --- | --- | --- | --- |
| Initiate ventilation within 60sec  **0**= >60sec, **2**=Yes |  |  |  |  |
| Call for help & Place probe on right hand before connecting to monitor (Ask)  **0**=Not done / done incorrectly, **1**=Done with prompting, **2**=Done correctly |  |  |  |  |
| Initiate ventilation with FiO_2_ 21%  Ask for information of settings: **0**=FiO_2_>21%, **1**=FiO_2_ 21% |  |  |  |  |
| Select appropriate size face mask  **0**=Incorrect size, **2**=Correct size |  |  |  |  |
| Place mask on face so that a seal is formed over chin, mouth & nose  **0**=Incorrect, tightens mask on face, **1**=Inconsistent, **2**=Correct at all times |  |  |  |  |
| Check for effective ventilation & initiate MRSOPA within 15sec (10 – 15breaths)  **0**=Ineffective ventilation & no MRSOPA within 15sec, **2**=Effective ventilation ± MRSOPA within 15sec |  |  |  |  |
| Effective ventilation at rate of 40-60/min for 30sec (Ask)  **0**=<40/>60/min / incorrect duration, **1**=Inconsistent effective ventilation, **2**=Correct rate & duration |  |  |  |  |
| Evaluation: Respiration, HR, oxygenation  **0**=No / 1 of 3, **1**=2 of 3, **2**=All 3 (*No point for HR assessment via umbilical pulsation*) |  |  |  | **Subtotal**  15 |
| ***“Apnoeic,*** ***HR = 40/min, Pulse oximetry not detecting, Cyanosed”***  ***‘Effective ventilation has been achieved, what would you want to do next?’*** | | | |  |
| 1. **Chest Compression** | | | |  |
| Identify need for chest compression  **0**=No, **1**=With prompt, **2**=Yes |  |  |  |  |
| Call for help  **0**=No, **1**=Yes |  |  |  |  |
| ***“You perform the chest compression & let me know what to do”*** | | | |  |
| Mention increase in FiO_2_ to 100% when chest compressions **begin**  **0**=Did not increase FiO_2_ immediately / Increase <100%, **1**=Increase with prompt to 100%, **2**=Increase to 100% |  |  |  |  |
| Correct placement of both thumbs on lower ^1^/_3_ of sternum with hands encircling chest  **0**=Incorrect / use 2-finger / don’t encircle chest, **1**=Inconsistent, **2**=Correct |  |  |  |  |
| Coordinated 3 compression : 1 ventilation  **0**=Incorrect, **1**=Inconsistent, **2**=Correct |  |  |  |  |
| Compress ^1^/_3_ of AP diameter of chest with complete recoil & no thumb lift  **0**=Incorrect, **1**=Inconsistent, **2**=Correct |  |  |  |  |
| At least 45-60sec of coordinated chest compression (*Ask*)  **0**=Incorrect duration, **1**=Yes |  |  |  |  |
| Evaluation: Respiration, HR, oxygenation  **0**=No / 1 of 3, **1**=2 of 3, **2**=All 3 (*No point for HR assessment via umbilical pulsation*) |  |  |  | **Subtotal**  14 |
| ***If done correctly, HR >100/min, irregular respiration, SaO_2_ 80%*** | Total score | | | 40 |

Appendix IIb

**Skills Assessment Checklist (Final Assessment)**

Participant ID/Name: ________________________ Date of Assessment: ____________

Evaluator ID/Name: __________________________

**Scoring: 0**=Not Done, **1**=Done incorrectly, incompletely or out of order, **2**=Done correctly in order

| ***“As part of our evaluation of the e-simulated learning tool, we are reviewing whether the tool improves the key basic skills of neonatal resuscitation. When you begin the resuscitation, work as if this were a real baby & a real resuscitation. The mannequin neither has a pulse nor can change in color, so please ask me for additional information. If you require equipment beyond what is available to you, do inform us. If you require an assistant, indicate that you need help. I will be the assistant but please tell me what to do. Do you have any questions?”*** | | | |  |
| --- | --- | --- | --- | --- |
| **Scenario** | | | |  |
| ***“You are called to attend a precipitous delivery in the antenatal clinic. The mother has ruptured membranes and is about to deliver. She is 35yrs, G6P5 with a*** ***singleton pregnancy at 38 weeks. There are no other risk factors and the amniotic fluid is clear.”***  ***“The equipment has been checked.” (Evaluator to orientate participants on equipment available)***  ***“The baby is born with a tight cord around neck.”*** | | | |  |
| **Skills** | **0** | **1** | **2** |  |
| 1. **Initial Assessment & Initial Steps** | | | |  |
| Start timer  **0**=Not started / Started late, **1**= Started immediately |  |  |  |  |
| 4 questions - Term, Liquor, Tone, Crying/Breathing?  **0**=Does not ask Tone & Crying/Breathing, **1**=Ask at least Tone & Crying/Breathing, **2**=Ask all 4 |  |  |  |  |
| ***“ Term, Liquor clear, Limp, Apnoeic”*** | | | |  |
| Dry baby, Keep warm and Remove wet towel  **0**=Not done, **1**=Incomplete drying, not removed wet towel, **2**=Perform both  (*No penalty for not mentioning “pre-warmed’*) |  |  |  |  |
| Position airway  **0**=Neck flexed / hyperextended, **2**=Correct position |  |  |  |  |
| Suction mouth & nose  **0**=Not done, **1**=Incorrect order, duration, depth, **2**=Correct order, duration, depth |  |  |  |  |
| Stimulate by flicking soles &/or rubbing back  **0**=Not done / incorrect, **1**=Stimulate correctly |  |  |  |  |
| Evaluation: Respiration, HR  **0**=No/improper evaluation/Only 1, **1**=Both (*No point for HR assessment via umbilical pulsation*) |  |  |  | **Subtotal**  11 |
| ***“Apnoeic, HR = 80/min”*** | | | |  |

| 1. **Bag & Mask Ventilation** | | | |  |  |
| --- | --- | --- | --- | --- | --- |
| Initiate ventilation within 60sec  **0**= >60sec, **2**=Yes |  |  |  |  | |
| Call for help & Place probe on right hand before connecting to monitor (Ask)  **0**=Not done / done incorrectly, **1**=Done with prompting, **2**=Done correctly |  |  |  |  |  |
| Initiate ventilation with FiO_2_ 21%  Ask for information of settings: **0**=FiO_2_>21%, **1**=FiO_2_ 21% |  |  |  |  |  |
| Select appropriate size face mask  **0**=Incorrect size, **2**=Correct size |  |  |  |  |  |
| Place mask on face so that a seal is formed over chin, mouth & nose  **0**=Incorrect, tightens mask on face, **1**=Inconsistent, **2**=Correct at all times |  |  |  |  |  |
| Check for effective ventilation & initiate MRSOPA within 15sec (10 – 15breaths)  **0**=Ineffective ventilation & no MRSOPA within 15sec, **2**=Effective ventilation ± MRSOPA within 15sec |  |  |  |  |  |
| Effective ventilation at rate of 40-60/min for 30sec (Ask)  **0**=<40/>60/min / incorrect duration, **1**=Inconsistent effective ventilation, **2**=Correct rate & duration |  |  |  |  |  |
| Evaluation: Respiration, HR, oxygenation  **0**=No / 1 of 3, **1**=2 of 3, **2**=All 3 (*No point for HR assessment via umbilical pulsation*) |  |  |  | **Subtotal**  15 | |
| ***“Apnoeic,*** ***HR = 40/min, Pulse oximetry not detecting, Cyanosed”***  ***‘Effective ventilation has been achieved, what would you want to do next?’*** | | | |  | |
| 1. **Chest Compression** | | | |  |  |
| Identify need for chest compression  **0**=No, **1**=With prompt, **2**=Yes |  |  |  |  |  |
| Call for help  **0**=No, **1**=Yes |  |  |  |  |  |
| ***“You perform the chest compression & let me know what to do”*** | | | |  |  |
| Mention increase in FiO_2_ to 100% when chest compressions **begin**  **0**=Did not increase FiO_2_ immediately / Increase <100%, **1**=Increase with prompt to 100%, **2**=Increase to 100% |  |  |  |  |  |
| Correct placement of both thumbs on lower ^1^/_3_ of sternum with hands encircling chest  **0**=Incorrect / use 2-finger / don’t encircle chest, **1**=Inconsistent, **2**=Correct |  |  |  |  |  |
| Coordinated 3 compression: 1 ventilation  **0**=Incorrect, **1**=Inconsistent, **2**=Correct |  |  |  |  |  |
| Compress ^1^/_3_ of AP diameter of chest with complete recoil & no thumb lift  **0**=Incorrect, **1**=Inconsistent, **2**=Correct |  |  |  |  |  |
| At least 45-60sec of coordinated chest compression (*Ask*)  **0**=Incorrect duration, **1**=Yes |  |  |  |  |  |
| Evaluation: Respiration, HR, oxygenation  **0**=No / 1 of 3, **1**=2 of 3, **2**=All 3 (*No point for HR assessment via umbilical pulsation*) |  |  |  | **Subtotal**  14 | |
| ***If done correctly, HR >100/min, irregular respiration, SaO_2_ 80%*** | Total score | | | 40 | |
